# P-436. Anal Pap Cytology for Anal Intraepithelial Neoplasia in Individuals under 45 Years of Age

**DOI:** 10.1093/ofid/ofae631.636

**Published:** 2025-01-29

**Authors:** Irada Choudhuri, Alexander Layden, James Baran, Kevin B Faaborg, Deborah McMahon, Ken Ho, Yijia Li

**Affiliations:** University of Pittsburgh Medical Center, Pittsburgh, Pennsylvania; University of Pittsburgh Medical Center, Pittsburgh, Pennsylvania; UPMC, Pittsburgh, Pennsylvania; UPMC Presbyterian, Pittsburgh, Pennsylvania; University of Pittsburgh, Pittsburgh, Pennsylvania; University of Pittsburgh, Pittsburgh, Pennsylvania; University of Pittsburgh, Pittsburgh, Pennsylvania

## Abstract

**Background:**

Human Papilloma Virus (HPV)-related high-grade anal intraepithelial neoplasia (AIN) is the precursor of anal cancer. There are limited data identifying factors that influence AIN progression in adults eligible to HPV vaccines.

**Figure 1.**
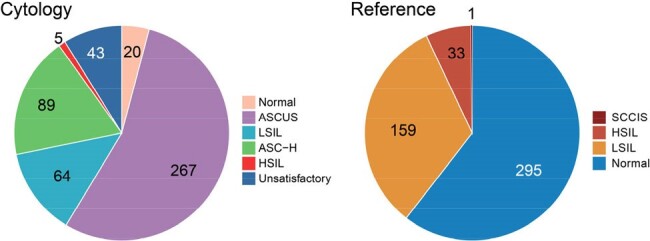
Distribution of anal cytology and reference test results (n=488). (A) Anal cytology. (B) Reference test ASCUS, atypical squamous cells of undetermined significance; LSIL, low grade squamous intraepithelial lesions; ASC-H, atypical squamous cells cannot exclude high-grade squamous intraepithelial lesions; HSIL, high-grade squamous intraepithelial lesions; SCCIS, squamous cell carcinoma in situ.

**Methods:**

This retrospective study includes individuals followed at University of Pittsburgh Anal Dysplasia Clinic from 2020-2024. Inclusion criteria were ages 18-45 years as of 2020, all sexes/genders, people with HIV (PWH) or without, and at least one anal cytology paired with pathology (reference standard) or high-resolution anoscopy (HRA) appearance (reference standard if pathology unavailable). We used Logistic regression to evaluate factors associated with high-grade squamous intraepithelial lesion (HSIL) or squamous cell carcinoma in situ (SCCIS) on reference tests (designated as ref-HSIL here). The University of Pittsburgh IRB approved this study.

**Table 1.**
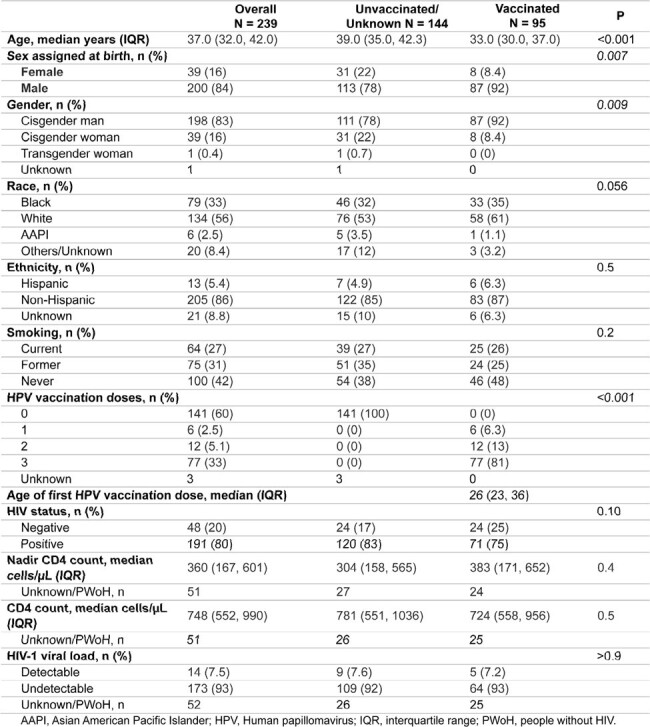
Baseline demographics.

**Results:**

We included 239 individuals, with median age 37 years, 16% female, and 95 (40%) received at least one dose of HPV vaccine at initial median age of 26 years (Table 1). Of 191 PWH, 93% were virologically suppressed, with median CD4 count 748 cells/µl at initial visits (2020 or after), and median nadir CD4 count 360 cells/µl. Distribution of cytology and reference tests (149 from pathology and 339 from HRA) was shown in Figure 1. Anal cytology had low-moderate sensitivity (55%) and specificity (82%), but an acceptable negative predictive value of 96% for ref-HSIL. In full cohort, risk factors for ref-HSIL were ASC-H (atypical squamous cells, cannot exclude HSIL) or HSIL on cytology (adjusted odds ratio [aOR] 6.19, P< 0.001). In PWH, nadir CD4 count <200 cells/µl (aOR 4.08, P=0.008) was associated with ref-HSIL while viral suppression tended to be protective (Table 2). Seven individuals with initial non-ref-HSIL progressed to ref-HSIL. Nadir CD4 < 100 cells/µl (aOR 25.0, P=0.010) and current smoking (aOR 6.2, P=0.095, Table 3) showed a trend in association with progression.

**Table 2.**
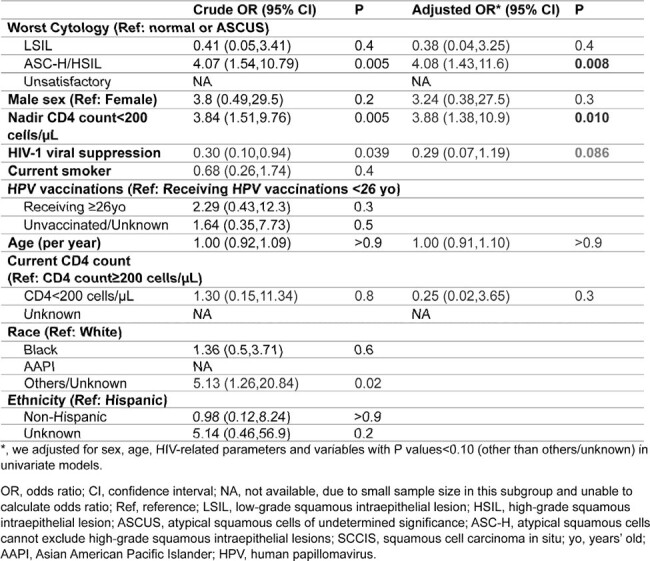
Factors associated with HSIL or SCCIS on reference tests in PWH.

**Table 3.**
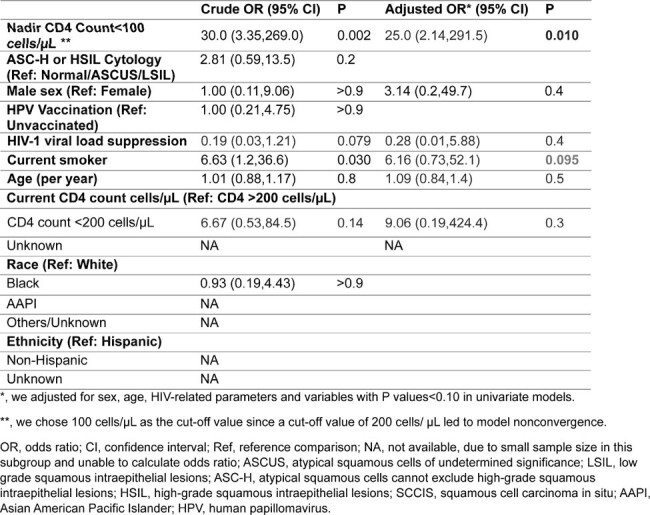
Factors associated with progression to HSIL/SCCIS.

**Conclusion:**

In this cohort of adults ≤45 years, cytologic ASC-H or HSIL predicts ref-HSIL. In PWH, low nadir CD4 count is a risk factor for ref-HSIL, while viral suppression is protective. Low nadir CD4 count is also a risk factor for AIN progression to ref-HIL. Our study helps prioritize target individuals who need HRA the most.

**Disclosures:**

**All Authors**: No reported disclosures

